# Transcranial Direct Current Stimulation (tDCS) Induces Adrenergic Receptor-Dependent Microglial Morphological Changes in Mice

**DOI:** 10.1523/ENEURO.0204-19.2019

**Published:** 2019-09-17

**Authors:** Tsuneko Mishima, Terumi Nagai, Kazuko Yahagi, Sonam Akther, Yuki Oe, Hiromu Monai, Shinichi Kohsaka, Hajime Hirase

**Affiliations:** 1 Laboratory for Neuron-Glia Circuitry, RIKEN Center for Brain Science, Wako 351-0198, Japan; 2 Brain and Body System Science Institute, Saitama University, Saitama 338-8570, Japan; 3 Center for Translational Neuromedicine, Faculty of Health and Medical Sciences, University of Copenhagen, 2200 Copenhagen, Denmark; 4 Faculty of Core Research Natural Science Division, Ochanomizu University, Tokyo 112-8610, Japan; 5 National Institute of Neuroscience, National Center of Neurology and Psychiatry, Kodaira 187-0031, Japan

**Keywords:** iba1, *in vivo*, microglia, norepinephrine, tDCS, two-photon imaging

## Abstract

Transcranial direct current stimulation (tDCS) has been reported for its beneficial effects on memory formation and various brain disorders. While the electrophysiological readout of tDCS effects is subtle, astrocytes have been demonstrated to elicit Ca^2+^ elevations during tDCS in a rodent model. This study aimed to elucidate the effects of tDCS on another major glial cell type, microglia, by histology and *in vivo* imaging. tDCS performed in awake conditions induced a significant change in the pixel intensity distribution of Iba-1 immunohistochemistry, and microglial somata were enlarged when examined 3 h after tDCS. These effects were blocked by adrenergic receptor antagonists or in IP_3_R2 (inositol trisphosphate receptor type 2)-deficient mice, which lack large cytosolic Ca^2+^ elevations in astrocytes. No obvious changes were observed in isoflurane-anesthetized mice. Furthermore, *in vivo* two-photon imaging of microglia showed a reduction of motility that was blocked by a β_2_-adrenergic receptor antagonist. Our observations add support for the influence of noradrenaline in tDCS and suggest possible interactions between microglia and astrocytes to express functional changes associated with tDCS.

## Visual Abstract

**Figure:**
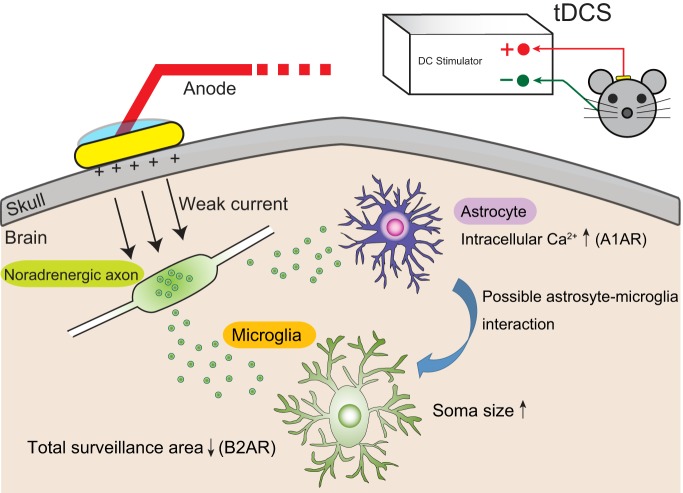

## Significance Statement

Transcranial direct current stimulation (tDCS) is a neuromodulation procedure in which a weak electric direct current is delivered through the brain for tens of minutes. Despite reported positive effects, the mechanisms of tDCS stimulation are not yet well understood. Here, we examined microglial morphology in the mouse cortex after tDCS. We find that the morphology and morphologic dynamics of microglia are altered by tDCS in a manner dependent on adrenergic receptors, supporting the notion that (nor)adrenergic signaling is involved in tDCS.

## Introduction

Noninvasive neuromodulation is a subject of intense research because of its potential for treating patients with neuropsychiatric and neurologic conditions. Transcranial direct current stimulation (tDCS) is the application of a constant and weak electric current to the brain through the skull. Typical parameters applied in humans are 1 mA over ∼30 cm^2^ for 10–30 min (Bikson et al., 2016). A fair sized body of published literature suggests that tDCS has positive effects on cognitive abilities and could be an alternative treatment for various brain disorders (Fregni and Pascual-Leone, 2007; Nitsche et al., 2008, 2009; Brunoni et al., 2012; Dedoncker et al., 2016). On the other hand, there is a notable degree of skepticism due to mixed outcomes of tDCS experiments (Horvath et al., 2015a,b; Jalali et al., 2017; Medina and Cason, 2017; Kunzelmann et al., 2018; Turkakin et al., 2018). The skepticism has been, in part, strengthened by a recent study that suggested negligible tDCS-induced membrane potential changes in cerebral cortical neurons (Vöröslakos et al., 2018), implying limited involvement of neuronal discharge as the prevalent mechanism of tDCS.

The circuit and cellular mechanisms for tDCS remain to be understood. Glial cells represent electrically nonexcitable cells in the nervous system. They have been regarded as “support cells” for the normal function of neurons. Among glial cell types, astrocytes and microglia maintain the extracellular milieu by ion homeostasis and phagocytosis, respectively. Additionally, astrocytes and microglia have been reported to interact with neuronal synapses (Wake et al., 2013; Araque et al., 2014). We recently reported that astrocytic Ca^2+^ surges occur during tDCS in mice. Moreover, tDCS-induced astrocytic Ca^2+^ surges were shown to promote cortical plasticity and have beneficial effects in a mouse model of depression (Monai et al., 2016; Monai and Hirase, 2016, 2018). The recruitment of Ca^2+^ activities in astrocytes has prompted us to investigate another major glial cell type, microglia.

Microglia are sensitive to brain tissue damage and transform to reactive microglia on inflammation. Iba1 (ionized calcium binding adaptor molecule 1) immunohistochemistry (IHC) visualizes the morphology of microglia, which is profoundly altered in reactive microglia. Following the published observation that reported the lack of pronounced microglial reactivity after tDCS (Monai et al., 2016), here we investigated Iba1 IHC in detail by digital image analysis. We report subtle, but significant effects of tDCS in an awake condition, but not under anesthesia, that depended on adrenergic receptors. Subsequently, we examined microglial motility by *in vivo* two-photon imaging and found that tDCS reduces microglial motility.

## Materials and Methods

All animal procedures were performed in accordance with the RIKEN animal experimental committee regulations.

### Animals

Adult C57BL/6J and IP_3_R2 (inositol trisphosphate receptor type 2) knock-out (KO) mice (Futatsugi et al., 2005) were used for immunohistochemical experiments (male, 2–4 months old). BAC-GLT1-G-CaMP7 line 817 mice (male, 2–5 months old; catalog #G7NG817, RIKEN BioResource Research Center; resource ID: RBRC09650) were used for transcranial macroscopic imaging of neuronal and astrocytic Ca^2+^ activity (Monai et al., 2016). Iba1-GFP mice (male, 3–10 months old; Hirasawa et al., 2005) were used for *in vivo* two-photon imaging of microglial morphology.

### Surgical procedures

Mice were deeply anesthetized with isoflurane (1.5–2.0%), and their scalps were exposed by shaving. Each mouse was ﬁxed on a stereotaxic apparatus (Narishige) under isoflurane anesthesia. Throughout the surgery and experiments with anesthetized mice, the body temperature was kept at 37°C with a heating blanket (BWT-100A, Bio Research Center). After topical application of xylocaine ointment (2% lidocaine) on the scalp, the skull above the sensory cortex was exposed by incision of the scalp and temporal muscle. A custom-made chamber ring was glued to the skull with cyanoacrylate superglue. After the glue settled, we applied dental cement (Fuji LUTE BC, GC Corporation; Super-Bond C&B, Sun Medical) for reinforcement. For two-photon imaging, the inner cavity of the chamber ring was reinforced with additional dental cement to secure the interface for an objective lens. Once the chamber ring was rigidly attached, the mouse was fixed on a custom-made stage via the chamber ring. Thereafter, a small craniotomy (φ = 3 mm, with intact dura) was carefully made using a dental drill.

### Habituation to head restraint

**Figure 1.**
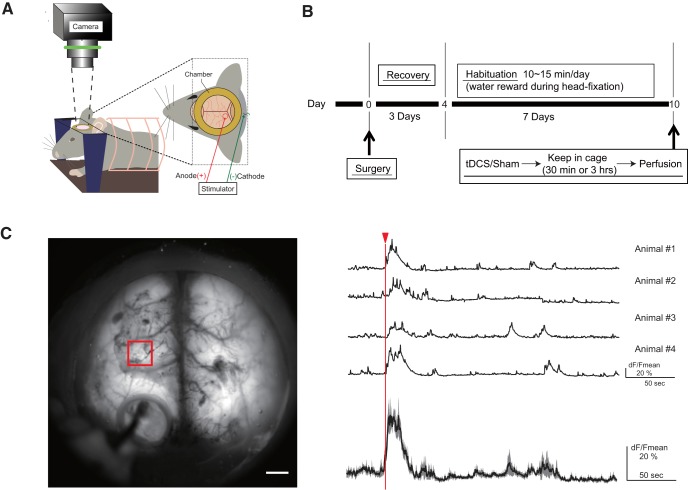
Head-restraint tDCS experiment. ***A***, Experimental setup for tDCS. ***B***, Experimental schedule of immunohistochemical experiment. ***C***, Top view of a BAC-GLT1-G7 Line 817 (G7NG817) mouse. Fluorescent Ca^2+^ signal is transcranially observable. Signals ∼3 mm anterior to the anodal site (1 × 1 mm^2^ red square) are plotted from four mice (right, top traces). The bold trace on the bottom is the mean of the four traces, and the shaded areas represent SE. The red arrowhead and line indicate the onset of tDCS. Scale bar, 1 mm. tDCS-induced Ca^2+^ elevations were not observed in isoflurane-anesthetized mice (Extended Data Fig. 1-1).

The postsurgical recovery period was at least 3 d for IHC experiments and 2 weeks for *in vivo* two-photon imaging experiments. Following the recovery period, mice were placed on a water restriction schedule and subjected to an acclimatization procedure for head restraint (Fig. 1*B*). Food was given *ad libitum*. The acclimatization procedure was performed for 7 d.

On day 1, each mouse was held in the experimenter’s hands and water was given via a syringe (∼0.2 ml). During handling, we let the mouse explore until it entered into a body tube similar to the one used with the tDCS apparatus. If the mouse entered the body tube, we repeated the procedure four to five times. The total handling time was 10 min for each mouse. From day 2, the mouse continued to be acclimatized to the experimenter and apparatus with a water reward (0.1–0.2 ml) for each entry to the body tube. At this point, the head of the mouse was quickly (<10–20 s) fixed to the apparatus via the chamber ring with its body in the tube. Additional water and sunflower seeds were provided during head fixation (10–15 min). The total amount of water given during head fixation was 1.0 ml/d. In some mice, for *in vivo* two-photon imaging acclimatization was performed for >7 d.

### Transcranial DC stimulation

tDCS was applied on mice under anesthesia (2% isoflurane) or in awake conditions. In either condition, the anode (stainless wire, φ = 350 μm) was placed on a sodium chloride-based conductive gel interface (Z101BA, NIHON-KODEN) spread over a circular area (φ = ∼2 mm) above the primary visual cortex (anteroposterior, −2.9 mm; mediolateral 2.0 mm). The cathode was connected to the neck skin after topical application of xylocaine ointment. DC (0.1 mA, 10 min) was applied with a custom-made isolated constant-current supply.

### Histology

After tDCS application, mice were kept for 30 min or 3 h before they were killed. After deep anesthesia by urethane, they were first perfused with 0.9% NaCl and later with fixative solution (4.0% paraformaldehyde in 0.1 m phosphate buffer, pH 7.4). Following brain removal and overnight postﬁxation in the same ﬁxative, coronal slices (60 μm) were prepared using a microslicer (PRO 7, Dosaka). For Iba1 staining, sections were incubated in a buffer containing the primary antibody (Tris-buffered saline with 0.1% Triton X-1000; 1:2000; catalog #019-19741, Wako) overnight. The sections were subsequently washed in PBS and incubated with the Cy3-conjugated secondary antibody (Invitrogen) for 2 h for ﬂuorescent labeling. To evaluate DSP4 [*N*-(2-chloroethyl)-*N*-ethyl-2-bromobenzylamine hydrochloride] efficacy, noradrenergic fibers were labeled by anti-tyrosine hydroxylase (TH) antibody (1:1000; catalog #AB152, Millipore) using sagittal slices (60 μm). For positive control of microglial reactivity, *Escherichia coli* lipopolysaccharide (LPS; 0.5 mg/kg) was administered by intraperitoneal injection 2 d before the mice were killed.

### Confocal imaging

Immunolabeled cortical microglia (V2 area) were examined using a confocal microscope (FV1000, Olympus). Images were acquired with a 60× water-immersion objective (UPlanSApo; numerical aperture, 1.20) at an excitation wavelength of 559 nm. Imaged areas covered 211.761 × 211.761 μm^2^ (1024 × 1024 pixels) with an optical sectioning of 0.5 μm. Images were scanned with the one-way mode (8 μs/pixel exposure).

### Drug administration

In some experiments, the following drugs were administered before tDCS by intraperitoneal injection: ICI81551 (5 mg/kg body weight, 30 min before; Tocris); and Prazosin (10 mg/kg, 30 min before; Sigma-Aldrich). For the ablation of noradrenergic neurons, DSP4 (Sigma-Aldrich) was injected 11 and 7 d before tDCS application (50 mg/kg, i.p., each time). Drugs were dissolved in 0.9% NaCl.

### *In vivo* imaging of microglial morphology

Adult Iba1-EGFP transgenic mice (Hirasawa et al., 2005), in which EGFP is expressed exclusively in microglia, were used to monitor microglial morphologic dynamics. All mice were habituated to the experimental apparatus for >7 d. On the day of imaging, the mouse was set on a custom-made stage under a two-photon microscope (B-Scope, Thorlabs). Microglia located >50 μm below the pial surface were imaged under awake conditions at a wavelength of 920 nm. The laser power was adjusted to ∼12 mW at the preparation (Hines et al., 2009; Wake et al., 2009; Pfeiffer et al., 2016). Depth stacks (24–26 slices, 2 μm *z* interval, 512 × 512 pixels corresponding to 101 × 101 μm^2^ or 201 × 201 μm^2^) were acquired every 60 s.

### Analysis

#### Iba1 IHC image analysis

Confocal images were used for pixel intensity analysis. Image stacks extending to 15 μm thickness were collapsed into 2D images by maximum intensity projection. Pixel intensities were converted to *z* scores, and the cumulative distribution was computed for each collapsed 2D image.

For soma size analysis, confocal image stacks (45–50 μm thickness) were ﬁrst ﬁltered with a 3 × 3 × 3 median ﬁlter. The resultant image stacks were collapsed into 2D images by maximum intensity projection. To correct for uneven background, the rolling ball method with a radius of 30 pixels was used for background subtraction. Thereafter, the images were subjected to a 3 × 3 2D median filter followed by binarization with Yen’s thresholding method (ImageJ, National Institutes of Health) for soma extraction. In some cases, manual adjustments of threshold were needed. Extracted somata were approximated to ellipses. Following these automated procedures in ImageJ, extracted somata were validated by manual inspection. The median of microglial soma size distribution from each mouse was taken as a data point for statistical comparisons.

#### TH image analysis

The efficacy of DSP4 was evaluated by calculating the mean intensity of posterior cortical layer 1 TH-positive (TH^+^) innervation using ImageJ. Briefly, sagittal brain section images (60 μm thickness) were acquired by a Keyence microscope (BZ-X710; 0.37 μm pixel size). Ten to 12 contiguous regions of interest (ROIs; 100 × 100 μm each) were allocated to occupy layer 1. A background intensity value was calculated from a neighboring parenchymal area that does not contain TH^+^ axons. The mean TH^+^ signal intensity of each ROI was computed as the mean pixel intensity minus the background intensity.

#### Microglial motility assessment

Quantification of microglial surveillance was performed using custom-written ImageJ and MATLAB programs (MathWorks). The maximum intensity projection image was computed for each time point of *xyzt* image stack. The resultant *xyt* image stack was registered for *xy* motion correction. Next, each slice of the *xyt* stack was processed by the ImageJ “Subtract Background” plugin to subtract smooth continuous background with a ball size of 30 pixels. Thereafter, images were treated with a 2D 3 × 3 median filter. After this preprocessing, rectangular areas containing the morphologic extent of single microglia were extracted as separate image stacks. These cell-wise image stacks were then binarized with a single threshold determined by Li’s Minimum Cross Entropy method (ImageJ). Noise reduction was then performed by a cycle of erosion and dilation. The normalized surveillance area at time *t* was calculated as the number of pixels that were occupied by the microglia at least once since the beginning of imaging until a given time *t*, divided by the number of pixels occupied by the microglia at the beginning. Normalized surveillance area is therefore a monotonically increasing function of time (see Fig. 6*C*,*D*, example). The surveillance index is defined as the ratio of normalized surveillance areas of a microglia in two different sessions [e.g., control (“Before”) vs post-tDCS (“After”)].

#### Statistical analyses

Statistical analyses were performed using Igor Pro (WaveMetrics). Student’s paired *t* tests and Wilcoxon-Mann–Whitney rank sum tests were used for the comparison of two sample populations with matched data and unmatched data, respectively, unless otherwise noted. Data are expressed as the mean ± SEM, and *p* values <0.05 were considered statistically significant. Statistical values are reported in Table 1.

**Table 1:** Statistical table

	Sample number: cells (animals)	Test type	*p* Value	Power
a	Sham: 334(7), Ctl: 315(7)	Mann–Whitney Wilcoxon rank sum test	0.1	
b	Sham: 315(7), tDCS: 314(7)	Mann–Whitney Wilcoxon rank sum test	0.16	
c	Sham: 309(7), tDCS: 301(7)	Mann–Whitney Wilcoxon rank sum test	**0.017	
d	Sham: 278(6), tDCS: 296(7)	Mann–Whitney Wilcoxon rank sum test	0.95	
e	Sham: 285(7), tDCS: 319(7)	Mann–Whitney Wilcoxon rank sum test	0.073	
f	Sham: 238(6), tDCS: 356(7)	Mann–Whitney Wilcoxon rank sum test	0.73	
g	Sham: 274(7), tDCS: 310(7)	Mann–Whitney Wilcoxon rank sum test	0.8	
h	Sham: 266(6), tDCS: 282(6)	Mann–Whitney Wilcoxon rank sum test	0.48	
i	Sham: 13(8)	Paired *t* test	0.82	0.055
j	tDCS: 11(8)	Paired *t* test	**0.014	0.77
k	Sham: 13(8), tDCS: 11(8)	Mann–Whitney Wilcoxon rank sum test	***0.006	
l	Sham: 11(3), tDCS: 9(2)	Mann–Whitney Wilcoxon rank sum test	**0.015	** **
m	Sham: 9(3), tDCS: 12(3)	Mann–Whitney Wilcoxon rank sum test	**0.023	

*p < 0.05, **p < 0.03, ***p < 0.01

## Results

First, we confirmed tDCS-induced cortex-wide Ca^2+^ elevations (Monai et al., 2016) in the present setting using G7NG817 transgenic mice that express the G-CaMP7 Ca^2+^ sensor in astrocytes and a subpopulation of neurons. Mice had been acclimatized to be rigidly fixed to a head-restraint platform, where tDCS (0.1 mA, 10 min) and transcranial fluorescence imaging were performed (Fig. 1*A*,*B*; see Materials and Methods). Cortical Ca^2+^ signals elevated immediately after the passage of the DC current. The peak amplitude of the G-CaMP7 response measured ∼3 mm anterior to the anodal position was 39.7 ± 4.1% (Fig. 1*C*; *N* = 4 mice), showing that tDCS-induced Ca^2+^ elevation is observable with the head chamber-ring configuration. Notably, tDCS-induced Ca^2+^ elevations were not observed in isoflurane-anesthetized mice (Extended Data Fig. 1-1). Having demonstrated the effectiveness of tDCS, we used C57BL/6 mice to investigate microglial morphology after tDCS by Iba1 IHC. Mice were killed either 30 min or 3 h after tDCS for perfusion fixation.

10.1523/ENEURO.0204-19.2019.ed1 Extended Data Figure 1-1 Cortical Ca^2+^ activity during tDCS in mice under deep isoflurane anesthesia. G-CaMP7 signal was transcranially measured from isoflurane-anesthetized (1.5–2.0%) BAC-GLT1-G7 Line 817 (G7NG817) mice. The top trace is for Sham stimulation (−3.14 ± 0.02%), and the lower trace is for tDCS (0.1 mA, 10 min; −4.30 ± 0.02%). Bold traces represent the mean of 11 traces from 9 mice. Shaded areas represent the SE. The red arrowhead and vertical line indicate the onset of tDCS or sham stimulation. Download Extended Data 1, EPS file.

### Iba1 IHC patterns are affected by tDCS in awake mice

**Figure 2.**
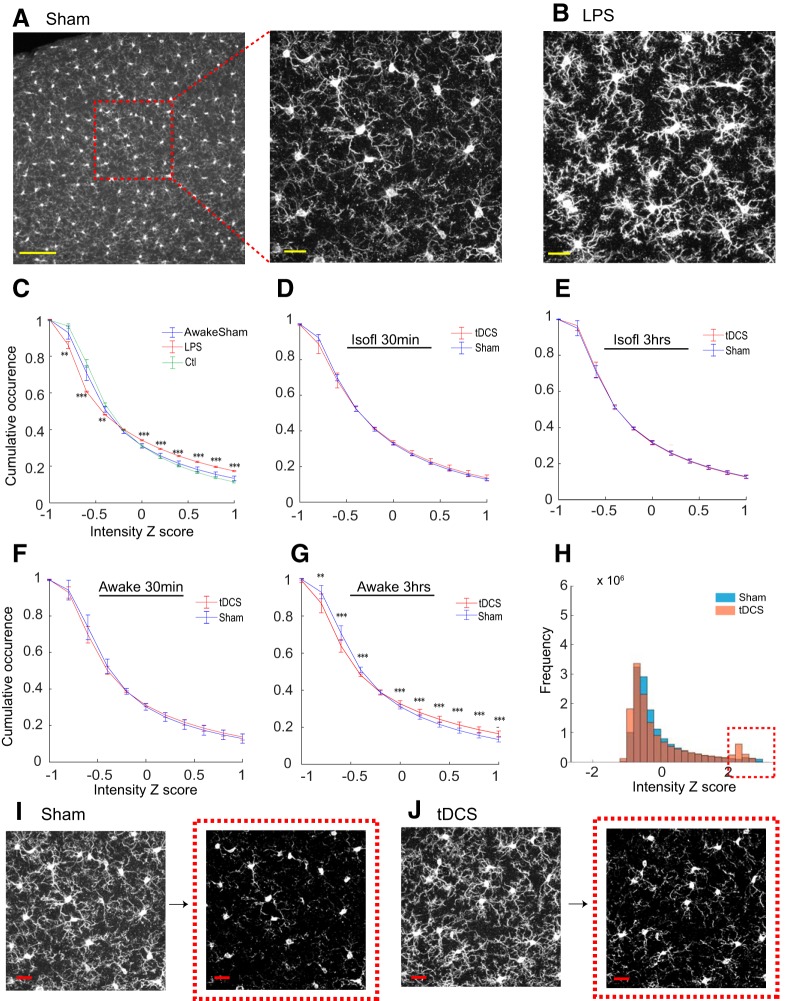
Intensity analysis of microglial confocal images. ***A***, ***B***, Representative images of Cy3-labeled Iba1 IHC by maximum intensity projection obtained in Sham- and LPS-treated mice. Yellow scale bars: ***A***, left, 100 μm; ***A***, right, ***B***, 20 μm. ***C***, Cumulative pixel intensity distributions from unrestrained Ctl and head-restrained Sham groups were similar and distinct from the LPS-treated group. ***D–G***, Intensity was compared between tDCS- and Sham-treated groups under the isoflurane-anesthetized (***D***, ***E***) or awake (***F***, ***G***) conditions, perfused at 30 min or 3 h after sham/tDCS. ***H***, In awake mice, the pixel intensity histogram indicates that there is a cluster at *z* score >2 (i.e., mean + 2 SDs) region in the tDCS group (dotted red square). ***I***, ***J***, Representative images from a sham-treated mouse and a tDCS-treated mouse. Images in the red squares correspond to the thresholded images on the left at the mean + 2 SDs. Red scale bars, 20 μm. **p < 0.01, ***p < 0.001.

Iba1 IHC visualized highly ramified microglial morphology throughout brain slices of sham-operated, LPS-treated, and tDCS mice (Fig. 2*A*,*B*,*I*,*J*). To investigate the impact of tDCS on the wide-field appearance of Iba1 IHC, we computed the pixel intensity distribution, which is a proxy of global morphologic changes. We analyzed layer 2 and 3 of the visual cortex located below the anode, since a previous study demonstrated that tDCS-mediated plasticity occurs in these layers (Monai et al., 2016). Pixel intensities were converted to *z* scores with which the cumulative distributions were plotted. We compared head ring-implanted, unrestrained control mice (Ctl group) with head ring-implanted, acclimatized, 25 min head-restrained mice (Sham group) to evaluate the possible effects of head restraint. In Figure 2*C*, we demonstrate that cumulative pixel intensity distribution is similar between the Ctl and Sham groups, whereas the pixel intensity distribution shifted significantly in mice with reactive microglia caused by LPS. These results suggest that the head-restraining procedure in acclimatized mice does not cause reactivity in cortical microglia.

Next, we compared tDCS and sham-treated mice. The combination of two conditions [isoflurane-anesthetized (isofl) or awake] and two time points (30 min and 3 h after tDCS) were investigated (Fig. 2*D–G*). Pixel intensity distribution was similar between sham and tDCS for isofl 30 min, isofl 3 h, and awake 30 min experiments; however, the awake 3 h tDCS data exhibited a visible deviation from the sham group (Fig. 2*G*). This deviation was caused by a higher proportion of pixels at the high-intensity end. For instance, tDCS had a relatively large presence of pixels that had a *z* score >0.6 (21.2 ± 1.6% vs 18.2 ± 1.4%; *p* = 1.7e-5, *t* test). Moreover, a high-intensity cluster that has a *z* score >2 was apparent in the pixel intensity histogram (Fig. 2*H*). Consistent with this observation, thresholding with *z* > 2 preserved more microglial structures in awake 3 h tDCS images than the sham counterpart (Fig. 2*I*,*J*). While the cumulative pixel intensity histogram of awake 3 h tDCS deviated in the same direction as LPS, microglial morphology appeared normal with fine ramified processes throughout the extent of the cortex in all tDCS experiments. Thus, tDCS does not appear to cause inflammatory responses.

### tDCS enlarges microglial somata in awake mice

**Figure 3.**
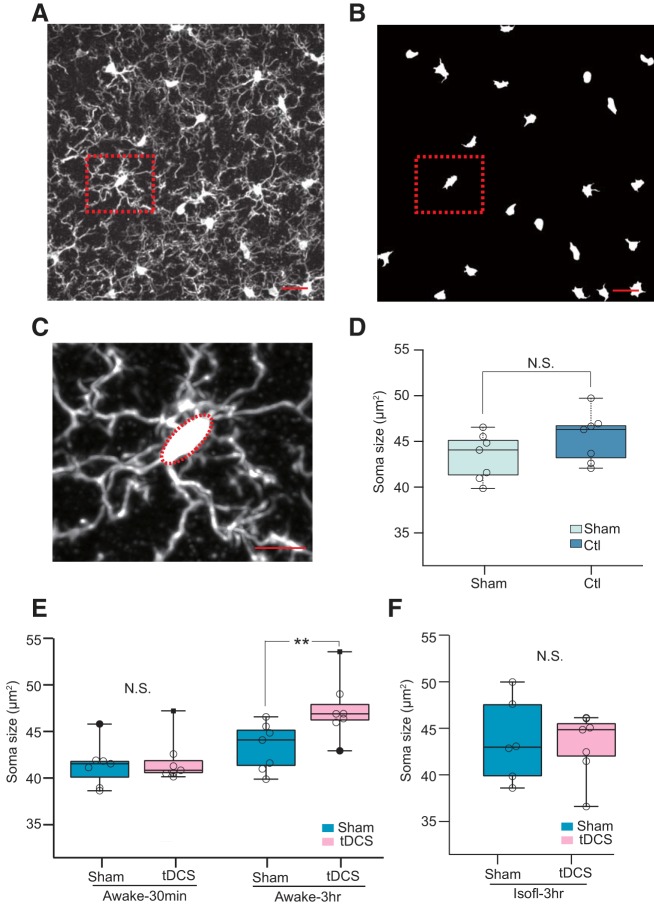
Quantification and comparison of microglial soma size. ***A***, Example image of an Iba1 IHC confocal image stack collapsed by maximum intensity projection. Scale bar, 20 μm. ***B***, Digitally processed image of ***A*** for soma extraction. ***C***, Example of the elliptic approximation of soma (***A***, ***B***, red dotted square). ***D***, Comparison of median values of microglial soma areas between Sham-stimulated and unrestrained control mice (*p* = 0.1^a^, Mann–Whitney Wilcoxon rank sum test). Scale bars: ***A***, ***B***, 10 μm; ***C***, 20 μm. ***E***, Comparison of microglial soma size in awake mice with/without tDCS treatment at different time points (30 min or 3 h) after tDCS. Microglial soma size was larger in the tDCS group in the awake 3 h experiment (*p* = 0.017^c^, Mann–Whitney Wilcoxon rank sum test). Each group contains seven mice. ***F***, Microglial soma size comparison in isoflurane-anesthetized mice (Isofl-3hr). **p < 0.03, N.S. not significant.

While the *z* score-based pixel intensity distribution analysis detected changes in the global appearance of images, it falls short of providing information on specific aspects of morphologic alterations. Microglial soma size has been reported to be sensitive to brain environmental changes (Kongsui et al., 2015). Therefore, we measured microglial soma size from Iba1 IHC images (Fig. 3*A–C*; see Materials and Methods). First, we compared the median microglial soma size of individual animals (43 cells per mouse on average) for unrestrained control and head-restrained sham groups as we did in Figure 2*C*. Figure 3*D* indicates that microglia soma sizes are similar between the control and sham groups (Ctl group: 45.4 ± 1.0 μm^2^, 7 mice; Sham group: 43.4 ± 1.0 μm^2^, 7 mice; *p* = 0.16^b^, Mann–Whitney Wilcoxon test), suggesting that the microglial soma size of the Sham group serves as a valid control for tDCS experiments.

Soma size did not differ significantly between the awake 30 min tDCS and Sham groups (Sham group: 41.5 ± 0.92 μm^2^, 7 mice; tDCS group: 41.9 ± 0.9 μm^2^, 7 mice; Fig. 3*E*; *p* = 1.0^a^). In awake 3 h experiments, soma size was significantly larger in the tDCS group (*p* = 0.017^e^; Sham group: 43.4 ± 1.0 μm^2^, 7 mice; tDCS group: 47.5 ± 1.2 μm^2^, 7 mice; Fig. 3*E*). On the other hand, soma size did not differ significantly when tDCS was performed on isoflurane-anesthetized mice (3 h group: *p* = 0.95^f^, Sham group: 43.7 ± 1.8 μm^2^, 6 mice; vs tDCS group: 43.2 ± 1.3 μm^2^, 7 mice; Fig. 3*F*). These results were consistent with the pixel intensity distribution analysis (Fig. 2) and suggest that isoflurane anesthesia hampers tDCS-induced microglial soma enlargement.

### tDCS-induced microglial soma enlargement is dependent on adrenergic receptors

**Figure 4.**
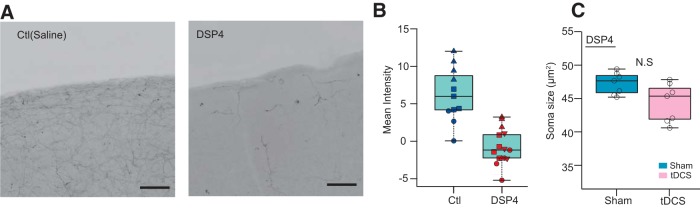
tDCS-induced microglial somatic enlargement depends on noradrenaline. ***A***, Example of cortical image (inverted grayscale) from saline- (left) or DSP4- (right) pretreated mice stained with TH antibody. ***B***, Mean intensity analysis of TH^+^ fiber. Each group contains data from three mice. Data from the same animals are plotted with the same symbol and color. Scale bars, 100 μm. ***C***, Comparison between median glial soma size from sham- and tDCS-treated mice (Sham group: 7 mice; tDCS: 7 mice; *p* = 0.073^e^, Mann–Whitney Wilcoxon rank sum test). N.S. not significant.

Recent human and animal studies have implicated the involvement of noradrenaline in tDCS (Monai et al., 2016; Kuo et al., 2017; Monai and Hirase, 2018; Souza et al., 2018). To examine the possible contribution of noradrenaline to tDCS-induced microglia soma size, we ablated noradrenergic cells in the locus ceruleus using the neurotoxin DSP4 (Bekar et al., 2008), which was confirmed by TH staining in the sensory cortex (Fig. 4*A*,*B*). Following noradrenergic neuron ablation, we performed tDCS using the awake 3 h protocol. As a result, DSP4-treated mice did not show a microglial soma enlargement after tDCS (Fig. 4*C*; Sham group: 47.3 ± 0.6 μm^2^, 7 mice; tDCS group: 44.5 ± 1.1 μm^2^, 7 mice; *p* = 0.073^e^).

**Figure 5.**
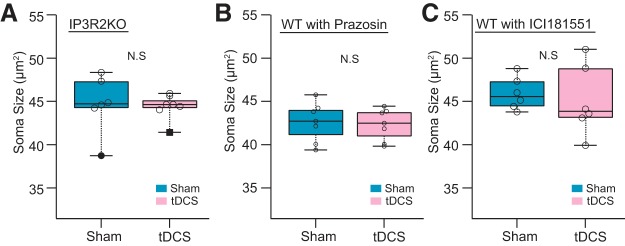
tDCS-induced microglial somatic enlargement depends on B2AR and A1AR pathways. ***A***, Comparison between median microglial soma size between Sham- and tDCS-treated IP_3_R2 KO mice (Sham group: 6 mice; tDCS group: 7 mice; *p* = 0.73^f^). ***B***, ***C***, Comparison of microglial soma size between Sham- and tDCS-treated wild-type strain C57BL/6J with prazosin (***B***; Sham group: 7 mice; tDCS group: 7 mice; *p* = 0.8 × *g*), or ICI181551 pretreatment (***C***; Sham group: 6 mice; tDCS group: 6 mice; *p* = 0.48^h^). N.S. not significant.

Since astrocytes exhibit profound α_1_-adrenergic receptor (A1AR)-mediated Ca^2+^ elevations by tDCS (Monai et al., 2016), astrocytic Ca^2+^ signaling possibly plays a role in the microglial soma enlargement via an intercellular communication. To examine this possibility, we used IP_3_R2 knock-out mice in which G_q_ GPCR (e.g., A1AR)-activated intracellular Ca^2+^ elevation is diminished in astrocytes. Awake 3 h tDCS did not result in significant microglial soma size changes in IP_3_R2 KO mice (*p* = 0.73^f^; Sham group: 44.6 ± 1.4 μm^2^, 6 mice; tDCS group: 44.4 ± 0.56 μm^2^, 7 mice; Fig. 5*A*). We next examined the involvement of A1AR using the specific antagonist prazosin in wild-type C57BL/6J mice. Similar to IP_3_R2 KO mice, prazosin-treated mice did not display tDCS-induced microglial soma enlargement compared with the Sham control group that also received the antagonist pretreatment (*p* = 0.8 × *g*; Sham group: 42.6 ± 0.9 μm^2^, 7 mice; tDCS group: 42.3 ± 0.7 μm^2^, 7 mice; Fig. 5*B*). These results suggest that tDCS-triggered noradrenaline release affects microglial soma enlargement via A1AR activation and the downstream astrocytic IP_3_R2-dependent Ca^2+^ signaling pathway.

Furthermore, we asked whether activation of β-adrenergic receptors is also involved. In particular, microglia are known for high levels of β_2_-adrenergic receptor (B2AR) expression (Tanaka et al., 2002, Gyoneva and Traynelis, 2013). Accordingly, mice were pretreated with ICI181551, a selective B2AR blocker, and soma sizes were compared. In the ICI181551 group, tDCS-induced soma size enlargement was not observed (*p* = 0.48^h^; Sham group: 45.9 ± 0.8 μm^2^, 6 mice; tDCS group: 45.2 ± 1.7 μm^2^, 6 mice; Fig. 5*C*). These results are indicative of noradrenergic involvement in tDCS-induced microglial changes and suggest that both A1ARs and B2BRs are involved in tDCS-induced microglial soma enlargement.

### tDCS decreases microglial surveillance area *in vivo*

**Figure 6.**
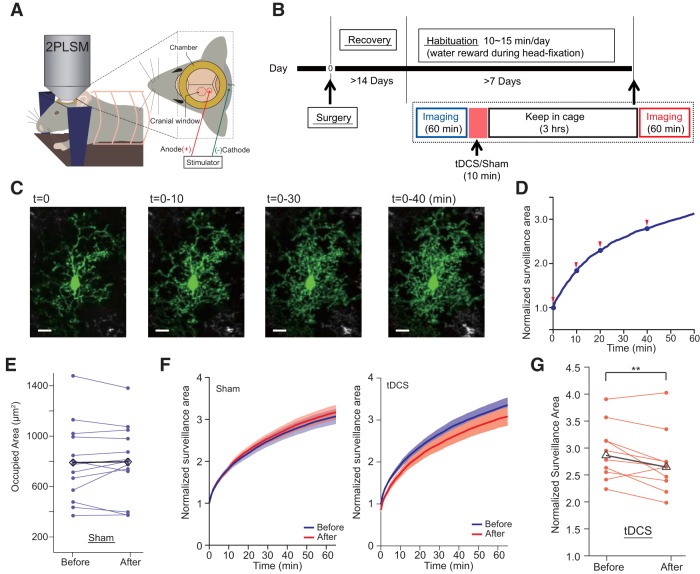
*In vivo* monitoring of microglial morphologic dynamics. ***A***, ***B***, Experimental setup (***A***) and time schedule of *in vivo* two-photon imaging (***B***). ***C***, Representative images of a microglia overlaid from *t* = 0 to respective time points (10, 30, and 40 min). ***D***, Normalized surveillance area curve during 60 min imaging period. Red arrowheads show the time points for the images in ***C***. Scale bar, 10 μm. ***E***, Initial microglial area at *t* = 0 of Before and After sessions are similar in Sham mice (13 cells from 8 mice; *p* = 0.82^i^). Blue lines represent data from individual microglia, and the black line represents averaged data. ***F***, Normalized surveillance area curves during the 60 min imaging period before (blue) and after (red) stimulation in the sham (left) and tDCS (right) mice. Data are represented as the mean ± SEM. ***G***, Normalized surveillance area at *t* = 40 min in Before and After sessions in tDCS-treated mice (normalized by surveillance area at *t* = 0/Before). Red lines represent data from individual microglia, and the black line represents averaged data. *p* = 0.014^j^, paired *t* test. **p < 0.03.

One of the striking features of microglia is the motility of their ramified processes. Here, we directly examined the morphologic dynamics of individual microglia in the cortex of awake mice using a two-photon microscope (Fig. 6*A*). We used the Iba1-EGFP mouse, in which EGFP is exclusively expressed in microglia (Hirasawa et al., 2005). We confirmed that microglia showed surveillance activities by continual extension and retraction of their processes in all directions (Davalos et al., 2005; Nimmerjahn et al., 2005). For example, the overlay of 60 min imaging resulted in an extensive coverage of the area within ∼60 μm from the soma, while the soma position remained unmoved (Fig. 6C*)*. We defined normalized surveillance area as the proportion of cumulative microglia-occupied area at a given time relative to a start time (Fig. 6*D*). To check whether laser scanning has an impact on microglial morphology, we compared the occupied area of each monitored microglia at the beginnings of Before and After imaging sessions of the Sham-treated group (Fig. 6*E*). We found no significant difference, suggesting that the effect of laser irradiation on microglial morphologic dynamics is negligible.

While the evolution of a normalized surveillance area varied considerably among individual microglia, the average trace converged to a gradually decelerating curve (Fig. 6*F*). The mean surveillance area after 60 min did not differ significantly between before and after sham stimulation. Remarkably, the mean surveillance area index curve of tDCS mice (i.e., After session) is plotted lower than the control condition (i.e., Before session). We assessed the surveillance area change of individual microglia by taking the ratio of the surveillance area indices during Before and After sessions, demonstrating a significant decrease of surveillance area by tDCS (*t* = 40 min, *p* = 0.014^j^, paired *t* test; Fig. 6*G*).

**Figure 7.**
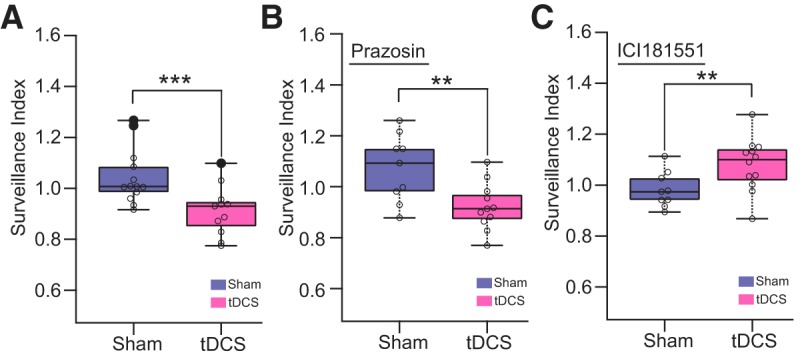
Microglial surveillance is compromised by tDCS. ***A***, Surveillance index at *t* = 40 min after sham/tDCS treatment in no drug-treated animals (Sham group: 13 cells from 8 mice; tDCS group: 11 cells from 8 mice; *p* = 0.006^k^). ***B***, Surveillance index comparison in prazosin pretreated mice (Sham group: 9 cells from 2 mice; tDCS group: 11 cells from 3 mice; *p* = 0.015^l^). ***C***, Surveillance index comparison in ICI181551-pretreated mice (Sham group: 9 cells from 3 mice; tDCS group: 12 cells from 3 mice; *p* = 0.023^m^) Mann–Whitney Wilcoxon rank sum test. **p < 0.03; ***p < 0.01.

Furthermore, we addressed whether noradrenergic signaling is involved in this tDCS-induced microglial surveillance reduction by prazosin or ICI181551 pretreatment in awake mice (Fig. 7*A*). As a reference, we computed the surveillance index comparing Before and After sessions at 40 min after the start of respective sessions. As expected from the previous analysis (Fig. 6*G*), the surveillance index of tDCS experiments was significantly reduced (Fig. 7*A*). Prazosin-treated mice showed a similar significant reduction of surveillance index after tDCS (Fig. 7*B*). By contrast, ICI181551 treatment abolished tDCS-induced reduction of microglia surveillance, and a trend for increased surveillance was apparent (Fig. 7*C*). These results point to a significant role of the B2AR in the inhibition of microglial surveillance activity after tDCS.

## Discussion

The present experiments report that tDCS induces subtle, but significant, alterations of Iba1 distribution and microglial motility in the cerebral cortex in awake mice. Furthermore, these alterations were dependent on (nor)adrenergic receptors, which is in line with the results of an earlier study that described tDCS-induced A1AR-dependent astrocytic Ca^2+^ surges (Monai et al., 2016). Notably, while astrocytic Ca^2+^ responses occur during tDCS, morphologic alterations of microglia occurred after a few hours.

We demonstrated that the microglial soma is enlarged after tDCS. Remarkably, the soma enlargement occurs only in awake mice. It is well established that microglial morphology is radically altered by LPS-induced inflammation (Kondo et al., 2011; Kozlowski and Weimer, 2012; Kongsui et al., 2015). LPS-induced microglial alterations are obvious even with a low dosage of 100 μg/kg, whereby ∼20% soma enlargement has been reported in the prefrontal cortex (Kongsui et al., 2015). The tDCS-induced microglial soma enlargement of a mere several percentage points in the current study is relatively modest. Moreover, no obvious change was detected in ramified processes. As general anesthesia compromises astrocytic Ca^2+^ activation, in particular noradrenergically driven large-scale and synchronized Ca^2+^ surges (Thrane et al., 2012; Ding et al., 2013), microglial changes by tDCS conceivably depend on the elevated noradrenergic tone during awake states. On the other hand, some studies have reported significant changes in anesthetized mice that underwent tDCS. For instance, one study reported that enhancements of GFAP and brain-derived neurotrophic factor (BDNF) in anesthesia changed gene expression (de Souza Nicolau et al., 2018). Another study showed long-lasting antidepressive behavioral effects (Peanlikhit et al., 2017). However, these studies used stronger stimulation in terms of stimulus current, duration, and/or frequency. Moreover, the anesthesia condition used in the current study is deeper than that in the study by Peanlikhit et al. (2017). Considering the lack of astrocytic Ca^2+^ surges in this condition (Extended Data Fig. 1-1), our results support the involvement of volume-transmitted neuromodulators in tDCS.

A few studies have examined cortical microglia after tDCS. For instance, Rueger et al. (2012) reported that multisession tDCS of 5–10 d induced a mild sign of microglial activation as observed by an upregulation of Iba1 immunohistochemical signals. The current density used in the study by Rueger et al. (2012) was ∼150 A/m^2^, whereas that used in the current study is <30 A/m^2^. Considering the study by Gellner et al. (2016), which reported a microglial activation threshold of 30–50 A/m^2^ with light isoflurane anesthesia, it is conceivable that our experiments were performed in near-threshold conditions. The tDCS-induced microglial soma enlargement and Iba1 signal intensity distribution shift are different from the microglial morphologic alterations reported in a rodent model of electroconvulsive therapy (ECT), in which obvious reductions in process ramification and Iba1 expression occur (Jinno and Kosaka, 2008). The pronounced alterations of microglia by ECT are most likely caused by the high-intensity electric stimulation that induces seizures. By contrast, cortical neuronal discharge activity remains undisturbed by tDCS (Monai et al., 2016; Vöröslakos et al., 2018).

We find that tDCS-induced soma enlargement is dependent on noradrenergic signaling. Moreover, the prazosin and IP_3_R2-KO mouse (which lacks astrocytic Ca^2+^ surges) experiments suggest a key mechanism linked to A1AR activation. The previous reports of relative abundance of A1ARs in astrocytes over microglia (Hertz et al., 2010; Zhang et al., 2014) and A1AR-dependent tDCS-induced astrocytic Ca^2+^ surges (Monai et al., 2016) support the idea that astrocytic activation exerts effects on microglia. While this is intriguing, neither the prazosin nor the IP_3_R2 KO mouse experiment is cell type specific; therefore, it is possible that direct noradrenergic activation of microglia causes soma enlargement. Indeed, B2AR inhibition by ICI181551 also disrupted microglial somatic enlargement. Functional and transcriptomic evidence underwrites the enriched expression of B2ARs in microglia (Tanaka et al., 2002; Gyoneva and Traynelis, 2013; Zhang et al., 2014).

By imaging microglial morphology in awake mice, we found that tDCS attenuates microglial motility. This effect was also dependent on B2ARs, but not on A1ARs. The inhibitory effect of microglial B2ARs on motility is consistent with the *in vitro* observation by Gyoneva and Traynelis (2013) and recent *in vivo* observations in awake mice (Liu et al., 2019; Stowell et al., 2019). It is tempting to speculate that the brake on microglial surveillance creates an opportunity for relevant synapses to establish an initial stage of synaptic plasticity. Microglia have been demonstrated to be a source for BDNF (Parkhurst et al., 2013), a pivotal neurotrophin for synaptic plasticity and neurogenesis. Interestingly, tDCS upregulates *Bdnf* (de Souza Nicolau et al., 2018), promotes BDNF-dependent synaptic plasticity (Fritsch et al., 2010), and causes epigenetic modification to *Bdnf* genomic regions (Podda et al., 2016). It remains to be shown whether BDNF synthesis is promoted by (nor)adrenergic activation as is reported in astrocytes (Jurič et al., 2008). In addition to astrocyte–neuron interactions (Monai and Hirase, 2016; Cocco et al., 2018), our results advocate for the inclusion of microglia as a functional component of the tDCS mechanism via adrenergic receptor activation.

One of the limitations of the current study is the lack of microglia-specific molecular manipulations. While it remains undetermined whether the microglial changes observed in this study have causal roles for positive outcomes of tDCS, several groups have consistently reported inflammation-associated microglial soma enlargement (Chen et al., 2012; Kozlowski et al., 2012; Kongsui et al., 2015). Brain inflammation activates microglia and leads to the production of proinflammatory molecules such as TNF-α, IL-1β, and IL-6 (Hanisch, 2002). It is possible that these cytokines are involved in the synaptic plasticity induced by tDCS. For instance, it has been demonstrated that the glial TNF-α has a pivotal role in the regulation of homeostatic synaptic plasticity (Stellwagen and Malenka, 2006). Future studies should address the causal relationship, for instance by microglial B2AR knock-out mice combined with tDCS and behavioral performance.

## Synthesis

Reviewing Editor: Maiken Nedergaard, University of Rochester Medical Center Decisions are customarily a result of the Reviewing Editor and the peer reviewers coming together and discussing their recommendations until a consensus is reached. When revisions are invited, a fact-based synthesis statement explaining their decision and outlining what is needed to prepare a revision will be listed below. The following reviewer(s) agreed to reveal their identity: Marco Aurélio Romano-Silva, Takahiro Kato.

The two reviewers and I are in agreement that this is a potential novel and important observation and that only minor revisions are required.

Reviewer #1

There is a great body of literature associated to tDCS effectiveness, mostly applied through clinical trials showing cognitive and functional rescue in diseased patients or enhancement in healthy volunteers as well as for several pre-clinical studies. The key point of this article is focused on elucidating components for tDCS mechanism which may further explain why tDCS is effective in cognitive modulation. There has been a great demand for such kind of studies seen that there is much we don't know about tDCS. The current study successfully demonstrates microglial morphological and surveillance alterations induced by tDCS. Furthermore, it highlights possible receptors that were significantly shown to be involved in these results. Although the data does show us that microglia is somehow altered in tDCS, it fails to associate these alterations to an actual mechanism. None of the presented results for microglia nor the references to it actually demonstrate if such alterations are in fact associated to tDCS' mechanism, being it may, these alterations could even be discussed as a collateral effect of the electrical current being applied to the animal's brain. To further corroborate what the authors conclude in the last paragraph of the discussion, the microglia inhibition should be shown together with the elimination of tDCS' effects such as cognitive alterations (behavioral assessment) or plasticity alterations (electrophysiological assessment). I do think the study brings important results to the field and that the experiments were well conducted, but I also feel that the discussion should be reviewed.

Statistics I see no problems concerning data analysis.

Visual Abstract(Required) N/A

Extended Data N/A

Software Comments N/A

Concerns about human or animal subjects I have no concerns regarding experimental conduct nor ethical issues.

Multimedia Comments N/A

Comments to the Authors (Required) I have no major concerns regarding this study.

There are a couple of minor considerations that should be assessed.

Lines 68 - 80: By providing this information this study will further assist other researches in replicating or continuing this investigation.

1. Please refer to the stereotaxic coordinates of the electrodes as well as its dimensions and material.

2. What conducting medium was used between the electrode and the scalp?

Line 196:

1. Sentence: “Iba1 IHC visualized......brain slices of tDCS and sham-treated mice (Fig. 2A,B)” seems not to refer to the correct figures since fig. 2 A-B depict control and LPS microglial activation. I think the correct images are 2 I-J

Lines 204-206 Fig. 2C:

1. Significance is presented in text but not shown in figure 2C. I understand the difference is in the LPS group, which is working as a positive control group, visually I see that there is a difference, but I still think it should be indicated in the figure.

Discussion:

1. With the exception of Ca++ influx alterations in astrocytes which has been previously demonstrated to have a direct influence on tDCS' mechanism and effectiveness in the 2016 paper by Monai and collaborators, there is no actual evidence that the microglia itself have a direct involvement. This conclusion may be achieved by the following factors: The use Transcranial Direct Current Stimulation is mainly driven by the necessity of treating neuropsychiatric and neurologic diseases and the vast number of cognitive deficiencies associated to them, and with some additional attempts of cognitive enhancement. Although this paper does show effects evoked by tDCS, it fails to provide if these alterations are simply collateral effects of the electrical current being applied to the animal's brain or that these effects actually have any involvement in how tDCS is able to modulate cognition. By inhibiting possible associated receptors such as B2AR and A1AR they were able to modulate microglial response to the electrical current, but by not showing that doing so also inhibited classical behavioral alterations or plasticity, fails to convince on microglia's involvement. This study shows that microglia response happens at some point between 30 minutes and above or up to 3h, while no effect is seen for the tDCS isoflurane exposed group. There are a couple of articles in the body of literature demonstrating successful behavioral alterations in tDCS stimulated mice in spite of isoflurane activity (Peanlikhit, 2017). The same is seen for tDCS induced calcium events in astrocytes (Monai, 2016) and gene expression (Nicolau, 2018) alterations, in which both situations, animals were exposed to isoflurane. I do believe the study was well conducted and that data itself is important for future investigations, but I recommend that the authors better discuss the data seeing that these results may have no connection to tDCS' actual mechanism, considering that it may as well be a collateral effect. To do so further experiments would be necessary.

General Text:

1. There are some grammar mistakes and missing words in the text, I believe it is nothing that would blur text comprehension but I do suggest the authors review the text.

Reviewer #2

tDCS is mainly understood from the viewpoint of neuron and astrocytes. In this study, the authors has focused on microglia in the process of tDCS, which is very novel approach.

Statistics NA

Visual Abstract(Required) NA

Extended Data NA

Software Comments NA

Concerns about human or animal subjects NA

Multimedia Comments NA

Comments to the Authors (Required) Majority of studies regarding tDCS has been focused only on neurons and astrocytes, and this study has shown us a novel study approach to clarify the microglial roles in tDCS.

The authors have revealed that microglial somata were enlarged when examined 3 hr after tDCS, and these effects were blocked by adrenergic receptor antagonists or in IP3R2 (inositol trisphosphate receptor type 2)-deficient mice which lack large cytosolic Ca2+ elevations in astrocytes. In addition, they showed that tDCS decreases microglial surveillance area in vivo. Overall, this study has nicely been conducted with appropriate study designs. The following comments/suggestions will improve the impact of this study.

1) The authors have indicated the importance of astrocyte-microglia interaction in the tDCS. However, this study did not show us the impact of microglial changes on the therapeutic effects of tDCS. In the initial part of the introduction, they highlighted that tDCS has positive effects on cognitive abilities. However, in this study, this therapeutic aspect has been ignored. Do the microglial somata enlargement and/or decreased microglial surveillance induce positive effects of tDCS on cognitive functions? Cognitive tests will help to answer the above fundamental questions, which will enhance the impact of this study.

2) The authors have estimated the activities of microglia by iba-1 staining and two-photon imaging. What do the enlargement of microglial somata mean? Other activation profiles such as cytokine release or mRNA expression of microglial cells may help to understand what kinds of activations are induced by tDCS.
